# Body habitus considerations in US anatomical body donation programs—Perspectives and practices from program guidelines

**DOI:** 10.1002/ase.70129

**Published:** 2025-11-04

**Authors:** Soph Myers‐Kelley, Jenna Hagerty, Heidi Reis, Kerry Sewell, Sophie Orr, Maureen Helgren, Rebecca L. Pearl, Marisa Langton, Malli Barremkala

**Affiliations:** ^1^ Laupus Health Sciences Library East Carolina University Greenville North Carolina USA; ^2^ Liaison Librarian to the Brody School of Medicine East Carolina University Greenville North Carolina USA; ^3^ Department of Medical Education Thomas Jefferson University Sidney Kimmel Medical College Philadelphia Pennsylvania USA; ^4^ College of Health & Human Performance East Carolina University Greenville North Carolina USA; ^5^ Department of Orthopaedic Surgery University of Michigan Ann Arbor Michigan USA; ^6^ Frank H. Netter MD School of Medicine Quinnipiac University Hamden Connecticut USA; ^7^ Department of Clinical and Health Psychology University of Florida College of Public Health and Health Professions Gainesville Florida USA; ^8^ Drexel University College of Medicine Philadelphia Pennsylvania USA; ^9^ Oakland University William Beaumont School of Medicine Rochester Michigan USA

**Keywords:** anatomical donor programs, anatomy, anatomy education

## Abstract

Many US body donation programs impose restrictions on donations based on body size. To examine the body habitus criteria related to body weight, height, proportion, and other size factors in United States body donation programs (USBDP), 127 USBDP publicly available websites listed by the University of Florida were reviewed. Since this study focuses on publicly available website information, it may not capture the characteristics of all programs. Eleven percent of the USBDP websites with public‐facing criteria exclusively listed numeric restrictions, while 24.4% included both descriptive and quantitative (numeric) terminology. The majority of websites with public‐facing criteria (90.6%) imposed a weight‐related restriction, with 55.1% providing descriptive (i.e., non‐numeric) terms. The most common body mass index (BMI) cutoff (30 kg/m^2^) could disqualify some U.S. adults from donating, as the average BMI in the United States for adult females is 29.8 and for adult males is 29.4. Fourteen programs offered explanations of their descriptive or numeric weight/BMI restrictions. As language evolves to better describe and reflect body habitus, keeping up to date with best practice and community preferences may help when designing donor criteria. Public‐facing information about body habitus criteria for donation may limit the representativeness of body habitus used in anatomical education and research. The implications of this on anatomical education and weight bias in students require more research.

## INTRODUCTION

Human anatomy has the honor of being considered the origin of medicine, a distinction that acknowledges a controversial and complex history. Historically, bodies for anatomical study have not been procured in a way that aligns with modern ethical sensibilities.^1^ In 1968, the U.S. Uniform Anatomical Gift Act, adopted in some form by all 50 states, set general guidelines for making, amending, or revoking anatomical gifts for education and research.^2^ However, individual U.S. institutions or programs provide the guidelines on eligibility criteria for donors.^3^ Investigation of how body donation programs publicly communicate their donation eligibility is important for stakeholders (donors, academic and research workforce, and students of anatomy) to understand the potential implications of eligibility requirements.

While criteria for body acceptance vary among programs, common reasons for ineligibility include infectious diseases, traumatic injuries, prior autopsy, decomposition, or extreme body size ‐ whether due to obesity or emaciation.^4^ Many of these restrictions are based on concern for student and faculty safety or the need for the presence of all intact organs to facilitate student familiarity with whole‐body anatomy.^4^ The rationale for size and weight‐related criteria might be unclear to prospective donors if they are not explicitly stated on a program's website. If the program's public‐facing criteria are adhered to and limit the inclusion of large‐bodied donors beyond the needs of personnel and student safety, this limits medical and health profession students' exposure to the range of different body types in the anatomy laboratory that is representative of the population of their future patients.

While there is a need for more research concerning how anatomy labs and the diversity of donors might impact a student, weight bias has been documented among medical students. A survey of 4732 first‐year medical students reported that 74% and 67% of students demonstrated implicit (unconscious) and explicit (conscious) weight bias, respectively.^5^ It has been suggested that negative attitudes, beliefs, and behaviors regarding obesity are introduced early and perpetuated throughout medical training and clinical practice, as learners observe and emulate physicians who express similar attitudes.^5^, ^6^ In one example of an anatomical lab, students working with larger bodies found them to be disgusting, unhealthy, and difficult to work with, whereas students placed with thin bodies felt lucky. Some students were inspired to lose weight as they felt disgust with their own bodies after working with larger donors.^7^ It is unclear whether the inclusion of larger‐bodied donors, with the appropriate educational scaffolding from anatomy professors, could reduce weight bias, increase weight bias, or create neutral or more positive attitudes in students, and whether those attitudes would result in changed patient care for larger‐bodied patients.

To investigate weight and height criteria cited on United States' body donation programs (USBDP) websites, this exploratory study evaluated the prevalence and rationale for body habitus criteria. Body habitus is defined as “the physical characteristics of an individual and includes such considerations as physique, general bearing, and body build.”^8^ In most cases, this would concern height, weight, and body proportions. We analyzed public‐facing, online information from USBDP websites to identify specific criteria related to weight, BMI, height, and the language used to describe them. This study aims to (1) identify terminology used for USBDP as related to body habitus, (2) assess the prevalence of specific body habitus criteria in USBDP websites, and (3) compare the donor pool based on these criteria to the general U.S. population.

## METHODS

Body donation program websites within the U.S. were identified using the University of Florida's list of 141 USBDP websites.^9^ After duplicates and obsolete program websites were removed, this yielded a final sample of 127 websites for analysis.

A data collection form was developed in REDCap to systematically extract data from USBDP websites. Key variables included the USBPD names; institutional affiliation; the presence or absence of body habitus‐related terminology (e.g., obesity, BMI, pounds, weight, height); specific criteria related to body habitus for donation eligibility; any quantified BMI, height, or weight criteria; and stated justification for these criteria. See (Appendix S1) for the REDCap data collection form. The presence or absence of a public‐facing DEI statement was originally included in the form, but is not reported in this paper due to an absence of statements across all surveyed websites. Additionally, during the peer review phase, it was determined to be beyond the scope of this article.

Twenty to twenty‐two USBDPs were assigned to each of six study team members to extract data from websites. To ensure the accuracy of the method, 11% of websites were randomly selected for duplicate review and cross‐checked by an independent reviewer from the team.

Data cleaning and analysis were performed using Google Sheets (Mountain View, CA, USA) and Microsoft Excel (Redmond, WA, USA). This process involved recoding variables and extracting the qualitative terms related to body habitus. Data cleaning involved adding columns for upper and lower criteria, changing data from yes or no to 1 and 0, and removing duplicate programs that were initially missed. Basic descriptive statistical analysis was performed. Frequency calculations were conducted to determine the prevalence of body habitus‐related criteria, presence of justifications for these criteria, and specific numerical criteria (for weight, height, or BMI) mentioned on the websites. Visual representations of the data were generated using GraphPad Prism 9 (San Diego, CA, USA).

## RESULTS

The findings from 127 USBDP websites were synthesized to highlight how body habitus criteria are communicated relative to the average US adult population. Fourteen programs (11%) exclusively listed numeric restrictions, so results reflect only publicly available criteria.

**FIGURE 1:**
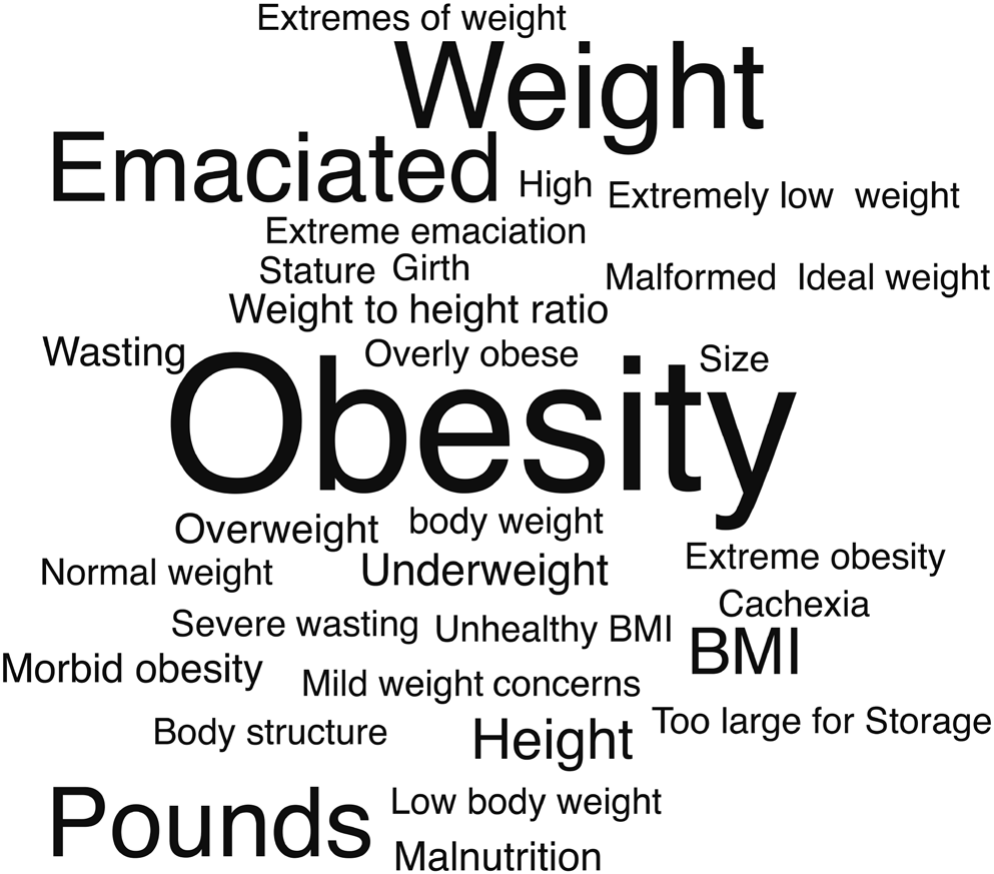
Word cloud of terminology used in US body donation program (USBDP) eligibility criteria related to body habitus. The size of each term corresponds to its frequency across program websites. The most frequent terms were obesity, weight, emaciated, and pounds.

A linguistic analysis of donor eligibility criteria revealed frequent use of terms describing weight and body size (Figure 1). The most common were obesity, weight, emaciated, and pounds. Terms related to height appeared less often, with height used most frequently and stature occasionally included. Expressions such as height‐to‐weight ratio, BMI, and unhealthy BMI appeared as well.

**FIGURE 2:**
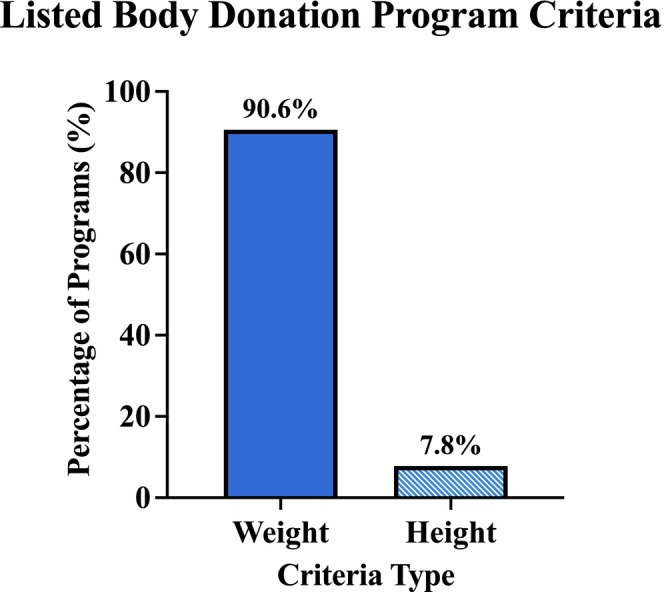
Proportion of USBDP websites that included weight‐ and height‐related donor criteria. Of public‐facing program websites, 90.6% (115/127) reported weight‐related restrictions and 7.9% (10/127) included height‐related restrictions.

Among websites sharing donor restrictions, 90.6% specified weight‐related criteria, and 7.9% included height‐related criteria (Figure 2). Programs provided either descriptive or quantitative limits—or both—for height and weight acceptability.

**FIGURE 3:**
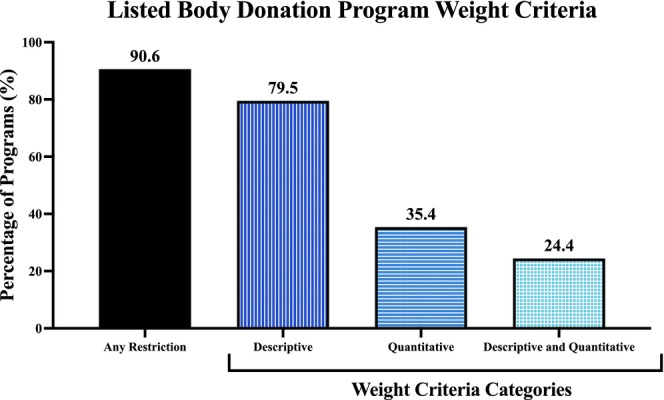
Categorization of weight‐related criteria by language type among public‐facing USBDP websites. Programs used descriptive terminology (79.5% or 101/127), quantitative thresholds (35.4% or 45/127), or both (24.4% or 31/127) to define eligibility.

Weight‐related criteria were more commonly described qualitatively than numerically (Figure 3). Descriptive terms appeared in 79.5% of programs, whereas only 35.4% used explicit numeric thresholds. Roughly, a quarter of programs (24.4%) incorporated both approaches, listing descriptive and quantitative terminology in their eligibility criteria.

**FIGURE 4:**
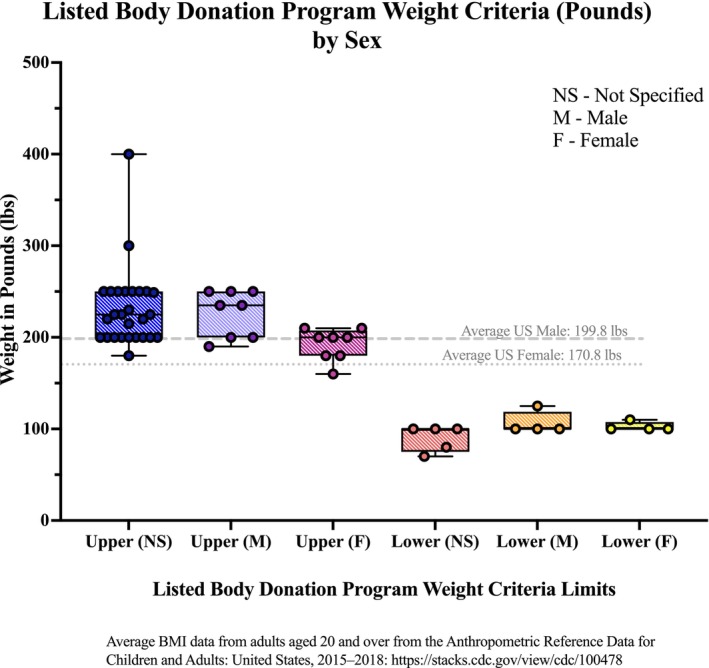
Distribution of upper and lower weight criteria reported by USBDPs, with and without sex differentiation. Sex‐specific upper limits ranged from 160 (female specific) to 400 lbs (not sex‐specific), and lower limits averaged 100 lbs (for both males and females) (±9.6 SD). US average adult weights are shown for comparison.^10^

Some programs published upper and lower weight limits differentiated by sex, while others applied non–sex‐specific (NS) thresholds (Figure 4). Among sex‐specific listings, the lowest upper weight criterion was 160 lbs for females, and the highest NS upper limit was 400 lbs. Lower limits ranged from 70 lbs (NS) to an average of 100 lbs for male and female (±9.6 SD). This figure also shows the average weight for males and females to provide context for how these weight criteria compare to the US adult population.^10^

**FIGURE 5:**
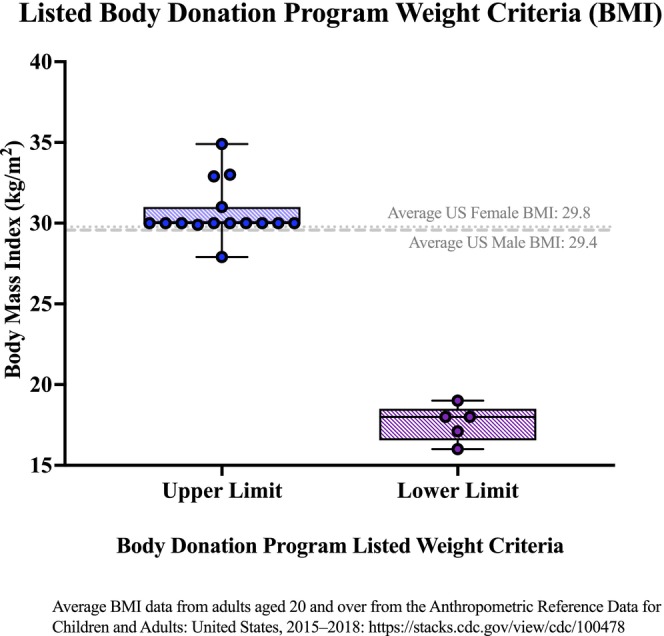
Upper and lower body mass index (BMI) criteria reported by USBDPs compared to national averages. Observed criteria were non–sex‐specific, with upper limits averaging 30 kg/m^2^ and lower limits ranging from 15–20 kg/m^2^.

Programs providing BMI criteria uniformly reported non–sex‐specific values (Figure 5). Upper BMI limits averaged 30 kg/m^2^, and lower limits ranged from 15 to 20 kg/m^2^. For comparison, national BMI averages are 29.4 for males and 29.8 for females.

Finally, qualitative review of the 14 programs that justified their weight and height restrictions revealed diverse language (Table 1). Explanations ranged from general descriptions of “weight problems” or “abnormal anatomy” to logistical constraints related to equipment or body preparation needs. Two thematic categories—general habitus‐based exclusions and practical/logistical exclusions—were identified to structure these justifications.

**TABLE 1:** Justifications for weight and height restrictions on 14 USBDP websites.

General justifications concerning body habitus	Justifications focused on equipment or logistical preparation of the body
A body that is significantly overweight or underweight is unsuitable for anatomical study; therefore, anatomical donors must fit within specific body mass index (BMI) restrictions at the time of death	For those donors DECLINED, it is due to stringent guidelines that allow the [program] to select only those donors that will remain well preserved and viable for prolonged study, and that are safe for our students to dissect
Or a weight problem that would prevent optimal use of the gift	The body is too large for storage in our storage area containers
Any condition that extensively destroys or distorts the normal anatomy of the body can make it difficult to conduct meaningful study	Due to equipment limitations
Ideal bodies for anatomical studies are within the normal weight range	Too large for storage purposes
Would prevent optimal use of the gift	Due to the nature of our preparation process, we are unable to accept donors weighing over 300 lbs
Body is not suitable for research and/or educational instruction	
Not suitable for health education purposes	
Purpose is to study normal human structure	
Our students need to study bodies not altered by major surgery or certain major diseases (author's note: major diseases including obesity)	

*Note*: Programs provided diverse rationales for excluding donors based on body habitus, categorized into (1) general body habitus justifications and (2) logistical or equipment‐related limitations. Program names are omitted for anonymity.

*Category definitions*:

- Quantified criteria: Defined as criteria providing a specific numerical value, such as weight in pounds or kilograms, a BMI in kg/m^2^, weight/height charts, or height in inches/feet.
- Descriptive weight criteria: Defined as a criterion that uses descriptive terms rather than numeric values to explain body habitus‐related exclusion. Examples include terms like emaciated, overweight, underweight, extremely low weight, obese, wasting, malnourished, overly obese, extreme obesity, and extremes of weight.

## DISCUSSION

Weight and BMI eligibility criteria posted on USBDP websites reveal significant variability, and the application thereof may exclude a substantial portion of the population from donating. Unlike a prior study conducted by Bagian et al., in which numerical maximum weights or BMIs were shared for 67 of the 72 survey responses of body program leaders,^4^ only 46 websites in our analysis listed specific numerical limits. The remaining 70 websites used vague description terms, leaving interpretation up to the donor—and potentially more leeway for the body donation program. The maximum weight allowed for donation varied widely from program to program, according to their publicly available websites. The maximum weight ranged from 160 lbs to 400 lbs, and BMI maximum weight limits ranged from 27–35. Boxplots revealed patterns with upper weight criteria for male donors spread between 200–250 lbs and upper weight criteria for female donors with a narrower spread and an average of 200 lbs. Upper BMI criteria averaged around 30 kg/m^2^. According to their criteria, only two USBDP programs accept donors weighing over 250 lbs. If programs adhere to their criteria, this naturally limits the number of eligible donors in the United States.

As a research team, we also wanted to learn what common justifications body donation programs shared publicly about this body habitus criteria. Of the 127 websites reviewed, 14 provided justification, citing equipment limitations and storage constraints or appropriateness for education. A few websites noted that larger bodies pose logistical challenges, particularly after the embalming process, which further increases the body's weight. The broader literature references students' and staff safety concerns when handling larger donors.^4^, ^11^, ^12^ Nine justifications on USBDP websites claimed that higher‐weight individuals were unsuitable for educational purposes (further justifications can be seen in Table 1). Whether these claims were meant to be explicit or implicit, we could not ascertain from the website. Safety concerns related to embalming processes and added weight, appropriate and safe body storage, and body maneuvering during the educational process are legitimate.^4^ When larger bodies are in the anatomy lab, appropriate measures must be taken to balance the needs of the anatomical students, the body donors seeking inclusion in anatomical education, and the financial limitations of the program. Larger equipment, training on body moving in teams, and reasonable body size restrictions with consideration to the current equipment's capacities can be considered, though specific instruction on inclusive best practices is beyond the scope of this paper.

When referring to public‐facing body donation practices, the language used to describe weight limits on these websites is not always in compliance with recommendations from research.^4^ Looking at a systematic review examining preferences for weight‐related terminology, findings recommend the use of neutral terminology like “weight” or “unhealthy weight”.^13^ The terms “obese” and “fat” were viewed overall as the least acceptable terms across studies. “Large size” and “heaviness” were negatively ranked as well. The term “BMI” offered conflicting results, some positive, some negative, and some neutral.^13^ Terms found on USBDP websites related to this commentary include BMI, morbid obesity, unhealthy BMI, extreme obesity, and overly obese. Some examples of language (see Table 1) found that may not meet language preferences and focus on the suitability for research include: “A body that is significantly overweight or underweight is unsuitable for anatomical study”, “a weight problem”, “not suitable for research and/or educational instruction”, “not suitable for health education purposes”.

The American Association for Anatomy's Human body donation programs best practices and recommended standards offer clearer recommendations in reference to the use of BMI terminology: “It is important to note that while BMI is widely used in healthcare, it is a calculation derived from data that does not represent diverse populations including people of color and females. BMI use can be inaccurate and discriminatory. However, an understanding of body composition may be needed to serve as a guideline to determine suitability for specific education or research activities, and BMI may be useful for this purpose. Body donation programs should strive to use alternate body composition markers whenever possible.”^14^ While body composition information may be helpful or even necessary when working with a body donor, the use of other markers is preferred. When possible, use weight and height instead of BMI as part of body donation criteria.

Some terms used on USBDP websites that align more closely with recommendations include extremes of weight, extremely low weight, ideal weight, overweight, body weight, normal weight, underweight, mild weight concerns, and low body weight. We noted that justifications focusing on storage or equipment limitations were able to avoid language that might be stigmatizing to donors entirely. Some examples from USBDP websites that align more closely with language preferences include: “The body is too large for storage in our storage area containers”, “Due to the nature of our preparation process, we are unable to accept donors weighing over 300 lbs”, and “For those donors DECLINED, it is due to stringent guidelines that allow the [program] to select only those donors that will remain well‐preserved and viable for prolonged study, and that are safe for our students to dissect”. As Puhl's systematic review concludes, while there is no universal recommendation on which words to use and which to avoid, there is general consensus that can be used as guidance.^13^

Medical and health professional education plays a critical role in addressing weight bias, yet effective strategies for reducing this bias remain underdeveloped. The potential of incorporating larger bodies into anatomy instruction while effectively mitigating bias is no different. According to a systematic review concerning weight bias reduction in health professionals, “there is no clearly defined approach to reduce weight bias among students and professionals in a health discipline”.^15^ However, there are some recommendations to discuss and consider. Some recommendations include revising the health professional curriculum, as pre‐professional education is a valued target for weight bias reduction.^15^ Phelan et al. suggest medical school curricula about disparities and stigmatized populations should include discussion about caring for patients with obesity. They also encourage incorporating positive experiences of medical students when placed in contact with a patient living with obesity (known as contact theory).^5^ It has been proposed that sharing positive experiences and hearing from role models who treat patients with obesity with respect and dignity could be influential for medical students in training.^11^ Whether a positive experience with a body donor who lived with obesity would influence medical students concerning weight bias reduction or not merits further research.

Body‐habitus‐related donation eligibility criteria vary greatly among institutions and are driven by end user requests. To reduce potential confusion across programs, we suggest that programs share the quantified maximum and minimum weight for body donation on their program websites. Evaluating equipment capacities, class capacities (considering safety), and the cost of chemicals and more adaptable equipment to ensure a safe student environment is also recommended. If financially feasible, expansions could be made to body habitus criteria to include larger and smaller bodies. Lastly, ensuring criteria language is up to date is critical in a rapidly changing landscape concerning body habitus description.

## LIMITATIONS

We believe the data presented in this study is a comprehensive collection of publicly available donation eligibility on USBDPs concerning body habitus. However, this dataset was created solely on information available publicly online. Only 11% of programs shared specific detailed weight restriction procedures. As such, this study cannot evaluate whether all programs are represented by this data, adhere to their procedures universally, or if they make exceptions for donors who are above or below their published weight and height limitations (and under what circumstances).

## FUTURE WORK

This research is exploratory and introductory. Further research could focus on analyzing the behavior of prospective donors seeking information online, whether the information donors find impacts their choice to donate, and what factors prevent some body donation programs from receiving enough donors. Research also needs to be done on how weight language and other language on public‐facing websites and promotional documents impacts prospective donors emotionally, and if it impacts their behavior.

Large‐bodied donors' educational impact in the lab and the best way to scaffold their integration in the classroom to positively impact students needs significantly more research. Similarly, as BMI rises in the United States, research concerning cost‐effective strategies to better incorporate large‐bodied donors would significantly benefit the anatomy educational community.

More research needs to be done to determine whether education in the anatomy lab impacts students' weight biases, and how that impacts their care of future patients of all sizes. How do professors communicate or model how to respect and work with larger bodies and what might a best practice or protocol entail?

Our research team is undertaking a survey of anatomy program workers and leaders on the fidelity of and rationale for weight restrictions of body donation programs. A follow‐up qualitative study will investigate the personal and institutional factors influencing body donation size limitations, focusing on the perspectives of those involved in program implementation.

## CONCLUSION

Anatomy courses are where health sciences students work with a human body on a deeply personal, sometimes years‐long venture. These anatomical gifts are a representation of the future patients they may treat. The findings of this research have many implications for the future of anatomical education and the beginnings of best practices for language describing body habitus criteria for USBDP donation. We hope this promotes respectful and intentional language describing the diversity of donors within the body donation process. May this inspire clearer ethical grounding in donation procedures and expanded incorporation of education into anatomy courses to the concept of working with a diverse range of bodies.

## AUTHOR CONTRIBUTIONS

**Soph Myers‐Kelley:** Conceptualization; data curation; investigation; methodology; project administration; writing – review and editing; writing – original draft. **Jenna Hagerty:** Data curation; writing – review and editing; writing – original draft. **Heidi Reis:** Data curation; investigation; validation; writing – review and editing; writing – original draft. **Kerry Sewell:** Conceptualization; investigation; writing – original draft. **Sophie Orr:** Data curation; formal analysis; visualization; writing – original draft; writing – review and editing. **Maureen Helgren:** Conceptualization; data curation; writing – review and editing. **Rebecca L. Pearl:** Conceptualization; methodology; investigation; writing – review and editing. **Marisa Langton:** Investigation; writing – original draft. **Malli Barremkala:** Conceptualization; data curation; investigation; methodology; writing – review and editing.

## CONFLICT OF INTEREST STATEMENT

All authors attest that they have no financial or other conflicts of interest.

## ETHICS AND INTEGRITY STATEMENT

All authors have done their best to adhere to research best practices in terms of ethics and integrity. No IRB approval was required due to a focus on publicly available website analysis. Living humans or body donors were not the subjects of data collection.

## Supporting information


**Appendix S1.** REDCAP Body Donation Website Analyzer.
