# Supplementary material for: Body habitus considerations in US anatomical body donation programs—Perspectives and practices from program guidelines
Source: Anat Sci Educ. 2025 Nov 4;19(1):85–92. doi: 10.1002/ase.70129 (PMC12748015; doi:10.1002/ase.70129)
Supplement: Supplementary file 1 — Appendix S1. REDCAP Body Donation Website Analyzer. [file ASE-19-85-s001.pdf]

Body Donation Website Analyzer

Please complete the survey as you evaluate body donation program websites.

Thank you!

Thank you for your help! If any question is not relevant to your example, please leave the question blank.

**Donation Program Name (the name, if any, of the program
associated with body donations)**

**Example with ECU's body donation program https://anatomy-cellbiology.ecu.edu/anatomical-gift-program/:
Donation Program Name: Anatomical Gift Program**

Is the program run by a college or university?

ECU Example: Yes

- Yes
- No

If it is associated with a college or university, what is the name of it?

Donation Program Homepage (URL)

ECU Example: https://anatomy-cellbiology.ecu.edu/anatomical-gift-program/

If there is no body donation program URL but there is a document with information about the program, please attach it here. If not, please skip.

ECU Example: Left blank

Does the body donation program website (or other information source) list requirements for donation?

ECU Example: Yes

* must provide value

- Yes
- No

Donation Program Requirements (URL)

ECU Example: https://anatomy-cellbiology.ecu.edu/suitability-of-donations/

Requirements for donation (if pastable)

ECU Example:
There are certain factors/conditions that may render the body unacceptable for an anatomical donation and some of these may not be obvious until the time of death. Examples of such factors/conditions that may invalidate an anatomical donation include:

the presence of certain infectious diseases by the deceased.
an autopsy on the deceased.
the presence of a major constriction or amputation of the limbs.
the weight of the deceased (taking into consideration the height and stature of the individual, the weight limit is approximately 170-180 pounds).

Requirements for donation (if attachment)

ECU Example: Left blank

Do any of these words appear on the site? (On homepage or subpage about body donation requirements, if applicable)

ECU Example:
Weight
Pounds
Other

- ☐ Obesity
- ☐ BMI
- ☐ Weight
- ☐ Pounds
- ☐ Other

What other body-mass-related keywords do you have?

ECU Example: Height, Stature

BMI? [preferably calculated variable based on weight/height]
>highest BMI accepted < lowest BMI accepted

ECU Example: Left blank

Do they list weight restriction?

ECU Example: Yes

* must provide value

- Yes
- No

Weight criteria (in pounds)

ECU Example: 170-180

Do they list height restriction?

ECU Example: No

* must provide value

- Yes
- No

Height (in feet & inches)

ECU Example: Left blank

"Is there justification for the weight requirements specifically for donation? (y/n)

ECU Example: No

* must provide value

- Yes
- No

What is the weight justification?

ECU Example: Left blank

Presence of DEI statement (Y/N)

ECU example: No

* must provide value

- Yes
- No

DEI Statement (if pasteable)

ECU Example: Left blank

DEI Statement (if attachment)

ECU Example: Left blank

Submit

Save & Return Later

Powered by REDCap - Cookie policy
